# Effects of genotype and environment on forage yield, nutritive value and morphology of lablab *(Lablab purpureus* (L.) sweet)

**DOI:** 10.1016/j.heliyon.2023.e19671

**Published:** 2023-08-31

**Authors:** Melkam Aleme, Metekia Tamiru, Ashraf Alkhtib, Getnet Assefa, Aemiro Kehaliew, Taye Tolemariam, Gezahagn Mengistu, Emily Burton, Geert Paul Jules Janssens

**Affiliations:** a Tepi Agricultural Research Centre, P.O. Box 34, Tepi, SNNPR, Ethiopia; b Jimma University, College of Agriculture and Veterinary Medicine, Department of Veterinary and Biosciences, PO Box 378, Jimma, Ethiopia; c Nottingham Trent University, School of Animal, Rural and Environmental Sciences, Brackenhurst Campus, Southwell, Nottinghamshire, NG25 0QF, UK; d International Livestock Research Institute, Land O'lakes (LOL), P.O. Box 64101 St. Paul, MN, 55164-0101, Addis Ababa, Ethiopia; e Holetta Agricultural Research Centre, P.O. Box 31, Holetta, Ethiopia; f Ghent University, Department of Veterinary and Biosciences, Heidestraat 19, B-9820, Merelbeke, Belgium

**Keywords:** Variety, Location, Forage, Lablab beans, Mixed farming system

## Abstract

The goal of the study is to determine the effect of genotype and environment on forage yield, forage nutritive value and to determine the relation between morphology and forage yield and nutritive value of lablab. Thirteen genotypes (one local and 12 improved) were replicated 3 times in a randomized complete block trial across three locations in Ethiopian lowlands namely, Bechi, Kite and Tepi. All forage samples were analyzed for dry matter (DM), crude protein (CP), and *in vitro* dry matter digestibility (IVDMD) using a combination of conventional nutritional analyses and near infrared reflectance spectroscopy. There was a significant (P < 0.001) effect of genotype, location and genotype*location on forage yield of DM, forage yield of CP, forage yield of IVDMD, CP, and IVDMD. The difference between means of minimum and maximum genotypes was 12.9 t/ha of DM, 3.12 t/ha CP, 8.22 t/ha IVDMD, 57 g/kg of CP and 56 g/kg of IVDMD. The correlation between plant morphology and forage yield and nutritive value was weak (r ≤ 0.41) in all locations and the combined data. Both genotype and location should be considered by the farmers when they decide to grow lablab for forage production. Morphological traits of lablab are not suitable to evaluate forage yield and nutritive value. Enhancing the awareness of farmers about the effect genetic-environment interaction effect of forage yield and nutritive value and the relation between morphology and yield and nutritive value would improve the uptake of lablab in mixed the farming system leading to more sustainable agricultural production.

## 1 Introduction

Crop-livestock mixed farming systems are the mainstay of smallholder livelihoods in the developing world [1]. The human population in mixed crop-livestock farming systems is increasing, resulting in high demand for food. The unplanned agricultural intensification and mono-cropping resulted in serious soil degradation, decline in natural species, and weed problems [2]. These changes lead to decreased in food supply to human and feed supply to livestock. Therefore, increasing food and feed supply without further degradation of the natural resources is required in mixed farming systems [3].

Integrating lablab *(Lablab purpureus* (L.) Sweet) in mixed farming systems could be a robust option for sustainable intensification [4]. Lablab is grown as an annual crop in Africa, South and central America, east and west Indies, China, south and south-east Asia and Australia for human consumption (grains) and livestock feeding (forage) [5]. Lablab is grown in a wide range of altitudes (0 m.a.s.l. to 3000 m.a.s.l), temperature (18 °C–35 °C), rainfall (650 mm–3000 mm) and soil types [6]. Introducing lablab to mixed farming systems would improve feed and food supply, increase soil content of nitrogen, break weed and disease cycles and decrease soil dehydration [5]. Lablab has high yield of grain (1 t/ha-5 t/ha). It is a summer growing legume; thus, it could supply the farm with high quality green forage (6 t/ha-9 t/ha; [5]) in the dry season when natural pasture is dry [7]. Lablab forage has high nutritive value as livestock feed containing high content of protein (CP) (125 g/kg DM-243 g/kg DM), low neutral detergent fibre (360–538 g/kg DM), low content acid detergent lignin (46 g/kg DM-107 g/kg DM), high calcium (7.4 g/kg DM-21.8 g/kg DM), high phosphorus (1.9 g/kg DM -5.5 g/kg DM) and high metabolizable energy (9.2 MJ/kg DM). Lablab forage has low levels of tannins (7.8 g/kg DM-21 g/kg DM) [5]. Supplementation of low-quality feed by lablab forage improves dry matter intake [8], milk production of cows [9] and growth of calves [10]. Goat fed low-quality basal diet had higher growth rate when supplemented with lablab hay [11]. [12,13].

The ILRI Genebank holds large set of lablab accessions (around 340 accessions) while about 200 of them were characterized using morphological and Amplified fragment length polymorphism markers. This characterisation showed large genetic diversity in the collection and enabled the development of a core collection [14]. Morphological variation in Lablab was assessed [15]. Intercropping lablab with cereal crops was investigated in sorghum [16], and maize [17]. The effect of fertilisation and accession on forage yield and nutritive value of lablab was determined [18]. There was a significant effect of genotype and location on agronomic traits of lablab [19]. However, no studies determined the effect of genotype and environment on yield and nutritive value of lablab forage.

Many studies assessed the relationship between morphology and forage yield and nutritive value in many crops in order to develop cheap and fast tool for determination of forage yield and nutritive value (for example [20] in faba bean [21], in chickpea). Yet, this was not assessed in lablab.

The goal of the current study is to determine the effect of variety and location on forage yield and nutritive value and to assess the correlation between morphological traits and forage yield and nutritive value of lablab.

## 2 Material and methods

### 2.1 Plant material and experimental layout

Twelve improved genotypes and one local genotype were obtained from International Livestock Research Institute (ILRI), Ethiopia and Bako Agricultural Research Centre, Ethiopia (Gebisa (local), ILRI110953, ILRI11612, ILRI11613, ILRI11614, ILRI11615, ILRI11619, ILRI14417, ILRI14425, ILRI14435, ILRI14445, ILRI14459 and ILRI6528). These accessions were bred by ILRI (recommended from a total of 98 ILRI genotypes) for forage yield and nutritive value and favourable agronomic traits in Ethiopian dry lowlands (Origin: Colina, Sao Paulo, Brazil; screen type: Experimental, year: 2015). The experimental genotypes were grown in three lowland locations, Kite, Tepi and Bechi during the main rainy season between July and February 2019 (Table 1). The locations vary in soil physical and chemical properties, temperature and rainfall. The experimental plots were manually planted at a rate of 20 kg/ha. The experimental plots were fertilised by di-ammonium phosphate at a rate of 100 kg/ha before planting. Hand weeding was applied 30 days after plantation at all experimental locations. The trials were replicated 3 times in the field with 4 rows per plot using randomized complete block design. The space between rows, plants, plots and blocks were 40 cm, 30 cm, 1 m, and 1.5 m, respectively. No irrigation was applied to any of the experimental plots. The plot size was 7.2 m (3 m × 2.4 m). The plots were prepared carefully to minimise the variability among blocks by applying proper hoeing and mixing to plots soil. Block was kept across the gradient. Sowing was done at the same time for all plots. Same weeding and agronomic practices were applied to all experimental plots.

**Table 1:** General description of the experimental locations.

Parameter	Kite	Bechi	Tepi
Longitude	35°51′ 469″E	35°55′ 467″E	35°25′ 05″E
Latitude	6°95′ 843″N	7°22′ 915″N	7°11′ 153″N
Altitude (m.a.s.l)	1200	1276	1200
Min temperature (ᵒC)	15.1	16.5	16.1
Max temperature (ᵒC)	27.5	35.3	30.2
Annual rainfall (mm)	1200	1574	1559
Soil analysis			
Soil type	Silt clay loam	Clay	Heavy clay
Soil texture			
Clay (%)	39	52	70
Sand (%)	21	19	17
Silt (%)	40	29	13
pH	5.1	5.9	6.3
P (%)	7.9	11.2	13.4
K (%)	227	495	528
N (%)	0.3	0.3	0.3
Organic carbon (%)	3.4	3.7	3.9
S (%)	12.5	12.5	12.5
Cation exchange capacity (m/equ/100)g soil)	0.1	0.14	0.2
Ca (g/kg)	1.69	3.03	3.23
Mg (g/kg)	0.257	0.411	0.439

All 5 cm above ground biomass of the two middle rows in each plot were harvested manually at the stage of flowering (when 50% of the plants flowered —after 107 days of seeding in average). The harvested forage for each plot were oven-dried at 65 ᵒC to constant weight and used to determine the forage yield. Subsamples (300 g) from each plot were ground to pass through a 1-mm mesh and stored for further nutritional analyses.

At the stage of flowering, 5 plants were randomly selected from each plot to measure plant morphology traits. Plant height was determined by measuring the distance between the ground and the tip of the flag leaf. Leaf area was measured by using photo electric leaf area meter GDY-500. At physiological maturity, the pods in all plots were manually picked off from the remaining rows to determine seed yield.

### 2.2 Forage quality analysis

The oven-dried forage samples were analyzed for crude protein, neutral detergent fibre and *in vitro* dry matter digestibility using a combination of conventional nutritional laboratory analyses and Near Infrared Reflectance Spectroscopy (NIRS; Instrument FOSS 5000 Forage Analyzer with WINSI II software package). A basal Near Infrared Reflectance Spectroscopy calibration was established and validated using conventional laboratory analysis of 20% of the samples. Nitrogen concentration was identified by Kjeldahl method using Kjeldahl (protein/nitrogen) Model 1026 (Foss Technology Corp.) (method 954.01 of [22]). The nitrogen content was multiplied by 6.25 to calculate crude protein. Neutral detergent fibre was determined according to Ref. [23] without using amylase in the determination and the result was expressed exclusive of residual ash. The *in vitro* dry matter digestibility was determined according to Ref. [24]. All forage nutritional analyses were done in Holetta Agricultural Research Centre Animal Nutrition Laboratory, Holetta, Ethiopia.

### 2.3 Statistical analysis

Data of the study was analyzed using analysis of variance according to the following model:

$$Y_{ijk}=\mu +G_{i}+{LO}_{j}+B{\left(LO\right)}_{jk}+G*{LO}_{ij}+{Ɛ}_{ijk}$$

Where Y_ij_ is the response variable, μ is the mean, G_i_ is the effect of genotype i, LO_j_ is the effect of location j, B(L)_kl_ is the effect of block k within location i, GLO_ij_ is the interaction between the variety and location and Ɛ_ijk_ is the residual. Least significant difference test at P = 0.05 with Bonferroni adjustment was used for mean comparison. The relationships between forage yield and nutritive value and morphology traits were calculated using Pearson's correlation. All statistical analysis were performed using R [25].

## 3 Results

### 3.1 Variability in morphology, forage yield and nutritive value

There were significant effects of genotype, location and their interaction on all study parameters (P < 0.001) (Table 2). There was a wide genotypic range in the combined locations (the difference between maximum and minimum genotype means) forage DM yield (11.5 t DM/ha), CP yield (3.12 t CP/ha), IVDMD (8.22 t IVDMD/ha), plant height (197 cm), leaf area (77.7 mm^2^), leaf to stem ratio (0.93), number of leaves per plant (73.9), number of pods per plant (24.1), and number of branches per plant (4.67) (Table 3, Table 4a, Table 4ba and 4b).

**Table 2:** Effect of genotype and location of forage yield of lablab.

	DM (t/ha)			CP (t/ha)			IVDMD (t/ha)		
Genotype	Bechi	Kite	Tepi	Bechi	Kite	Tepi	Bechi	Kite	Tepi
Gebisa (local)	6.81abc	3.13	6.13a	1.58abc	0.7	1.53a	4.15abc	1.92	3.96a
ILRI110953	10.1def	2.83	12.8de	2.44def	0.63	3.07bc	6.19def	1.72	8.04cd
ILRI11612	8.44bcde	3.02	10.8cd	2.03bcde	0.66	2.83bc	5.15bcde	1.8	6.97bc
ILRI11613	12.6f	3.08	14.2e	3.01f	0.72	3.45c	7.74f	1.85	9.05d
ILRI11614	4.7a	1.33	9.83bc	1.12a	0.33	2.69b	2.86a	0.83	6.33bc
ILRI11615	8.18bcde	1.83	10.1cd	1.97bcd	0.41	2.42b	4.96bcde	1.13	6.22b
ILRI11619	9.26cde	2.37	9.63bc	2.09cde	0.53	2.44b	5.54cde	1.44	6.2b
ILRI14417	7.37abcd	2	10.8cd	1.77abcd	0.46	2.77b	4.49abcd	1.23	6.89bc
ILRI14425	9.51cde	1.62	9.88bc	2.27de	0.38	2.5b	5.9de	0.98	6.25b
ILRI14435	5.7 ab	3.03	7.07 ab	1.36 ab	0.66	1.57a	3.52 ab	1.81	4.41a
ILRI14445	8.71cde	2.93	10.8cd	2.07cde	0.64	2.65b	5.31cde	1.78	6.78bc
ILRI14459	10.5ef	2	9.55bc	2.4def	0.46	2.49b	6.37ef	1.21	6.21b
ILRI6528	10.7ef	1.52	11.2cd	2.65ef	0.36	2.47b	6.61ef	0.93	6.95bc
Pooled SEM	1.02			0.24			0.62		
LSD	2.85			1.38			2.22		
ANOVA P value									
Genotype	<0.001			<0.001			<0.001		
Location	<0.001			<0.001			<0.001		
Genotype × Location	<0.001			<0.001			<0.001		

DM = dry matter; CP = crude protein; IVDMD = *in vitro* dry matter digestibility; SEM: standard error mean; means within a column with different letters are significantly different (P ≤ 0.05).

**Table 3:** Effect of genotype and location on crude protein and *in vitro* dry matter digestibility of lablab forage.

	CP (g/kg DM)			IVDMD (g/kg DM)		
Genotype	Bechi	Kite	Tepi	Bechi	Kite	Tepi
Gebisa (local)	229	223	250 ab	612abc	612	647c
ILRI110953	243	219	241 ab	605abc	605	631abc
ILRI11612	242	219	261b	597 ab	597	644bc
ILRI11613	239	233	243 ab	602abc	602	637abc
ILRI11614	236	249	274b	624c	624	643abc
ILRI11615	238	226	241 ab	616abc	616	623 ab
ILRI11619	230	224	253 ab	608abc	608	643abc
ILRI14417	240	230	260b	618bc	618	642abc
ILRI14425	239	234	257 ab	607abc	607	635abc
ILRI14435	239	217	221a	595a	595	622a
ILRI14445	237	219	249 ab	609abc	609	630abc
ILRI14459	231	230	262b	607abc	607	651c
ILRI6528	248	233	221a	609abc	609	622 ab
Pooled SEM	13.1			7.46		
LSD	10.2			7.9		
ANOVA P value						
Genotype	<0.001			<0.001		
Location	<0.001			<0.001		
Genotype × Location	<0.001			<0.001		

CP = crude protein; IVDMD = *in vitro* dry matter digestibility; SEM: standard error mean; means within a column with different letters are significantly different (P ≤ 0.05).

**Table 4a:** Morphological traits of 12 improved genotypes and one local genotype of lablab grown in three locations in Ethiopia.

	Plant height (cm)			Number of pods per plant			Number of branches per plant		
Genotype	Bechi	Kite	Tepi	Bechi	Kite	Tepi	Bechi	Kite	Tepi
Gebisa (check)	230b	119 ab	271a	28.5bcd	38.1a	19.3 fg	5.67 ab	5.07a	6.33bcd
ILRI110953	235b	132a	263abc	30.5abc	34.8abc	26.8cd	5.33 ab	5a	7.53 ab
ILRI11612	230b	105 ab	214d	22.6e	31.2bcd	14g	4.87b	4.87a	6bcd
ILRI11613	243 ab	111 ab	227bcd	32.8 ab	31.2bcd	32.8 ab	4.93b	4.53a	8.67a
ILRI11614	281a	84.2b	267 ab	30.2abc	29d	31.7abc	4.93b	4.93a	4.8de
ILRI11615	242 ab	114 ab	231abcd	23.8de	30.2cd	17.8 fg	5.2 ab	4.6a	7.13 ab
ILRI11619	226b	131a	214d	28.3bcd	36.8 ab	20.3ef	5.8 ab	5.27a	5.07de
ILRI14417	259 ab	108 ab	224cd	31.8 ab	34.2abcd	30.2abcd	5.8 ab	5.73a	4e
ILRI14425	229b	104 ab	217d	28.2bcde	31.1bcd	25.3de	6.73a	5.83a	5.8bcd
ILRI14435	226b	112 ab	235abcd	34.8a	36.6 ab	33.5a	6.13 ab	4.53a	6.4bcd
ILRI14445	238b	118 ab	263abc	25.7cde	35.5abc	16 fg	6.47 ab	4.6a	6.2bcd
ILRI14459	234b	115 ab	260abc	28.8bcd	30.6cd	27.4bcd	5.27 ab	5.13a	5.2cde
ILRI6528	252 ab	140a	252abcd	33.8 ab	32.3bcd	34a	6.93a	4.87a	6.87bc
Pooled SEM	14.9			2.04			1.14		
LSD	42			5.75			3.21		
P value									
Genotype	<0.001			<0.001			<0.001		
Location	<0.001			<0.001			<0.001		
Genotype × Location	<0.001			<0.001			<0.001		

SEM: standard error mean; means within a column with different letters are significantly different (P ≤ 0.05).

**Table 4b:** Morphological traits of 12 improved genotypes and one local genotype of lablab grown in three locations in Ethiopia.

	Leaf area (mm^2^)			Leaf to stem ratio			Number of leaves per plant		
Genotype	Bechi	Kite	Tepi	Bechi	Kite	Tepi	Bechi	Kite	Tepi
Gebisa (check)	65.3c	37.9a	71.3abc	1.73abc	1.2a	1.31c	62.4bc	59.2 ab	46.4de
ILRI110953	76.7bc	32.9a	75.2abc	1.53cd	1.33a	1.31c	55.1c	51.6abcd	40.3de
ILRI11612	72.1bc	34a	63.9bc	1.53cd	1.43a	1.14c	84a	43.4cde	39.7de
ILRI11613	83.9abc	31.7a	89.5a	1.9 ab	1.2a	1.5abc	63bc	63.4a	68.7b
ILRI11614	105a	31.5a	92.1a	1.87abc	1.19a	1.3c	52.1c	18.1g	61.7bc
ILRI11615	76.8bc	27.1a	80.3 ab	1.73abc	1.23a	1.33c	62.3bc	47.2bcde	92a
ILRI11619	73.9bc	32.9a	87.5a	1.77abc	1.2a	1.4bc	50.3c	44.1cde	37.8e
ILRI14417	80.4bc	36.4a	90.4a	1.73abc	1.48a	1.8a	72.6 ab	23.5 fg	46.3de
ILRI14425	69.4bc	29.2a	53.9c	1.37d	1.33a	1.71 ab	56.3c	34.8ef	52.3cd
ILRI14435	77.6bc	34.2a	88.1a	1.64bcd	1.23a	1.33c	53.1c	55.7abc	60.7bc
ILRI14445	69.1bc	33.5a	75.7abc	2.07a	1.3a	1.2c	78.3a	49.3bcd	49.5cde
ILRI14459	69bc	30.2a	76abc	1.53cd	1.4a	1.5abc	52c	41.8de	71.7b
ILRI6528	88.9 ab	31.3a	71.7abc	1.69bcd	1.3a	1.2c	55c	55.3abcd	43.3de
Pooled SEM	8.25			0.13			4.85		
LSD	23.3			0.366			13.7		
P value									
Genotype	<0.001			<0.001			<0.001		
Location	<0.001			<0.001			<0.001		
Genotype × Location	<0.001			<0.001			<0.001		

SEM: standard error mean; means within a column with different letters are significantly different (P ≤ 0.05).

ILRI11613, Gebisa, and ILRI110953 had the highest DM yield in Bechi, Kite, and Tepi, respectively. ILRI11613 had the highest CP yield in the three experimental locations. ILRI6528, Gebisa, and ILRI110953 had the highest IVDMD yield in Bechi, Kite, and Tepi, respectively. ILRI6528, ILRI14425, and ILRI11614 had the highest content of CP in Bechi, Kite, and Tepi, respectively. ILRI14435, ILRI14417, and ILRI14459 had the highest level of IVDMD in Bechi, Kite, and Tepi, respectively.

ILRI11614, ILRI6528, Gebisa had the heights plant height in Bechi, Kite, and Tepi, respectively. For leaf area, ILRI11614, Gebisa, ILRI11614 had the largest area in Bechi, Kite, and Tepi, respectively. The highest number of leaves per plant was found in ILRI11612, Gebisa, ILRI11615 in Bechi, Kite, and Tepi, respectively. The highest leaf to stem ratio in Bechi, Kite, and Tepi were found in ILRI14445, ILRI14417, and ILRI14417, respectively.

Number of branches per plant: ILRI6528, ILRI110953, ILRI11613 had the largest number of branches per plant in Bechi, Kite, and Tepi, respectively. The highest number of pods per plant in Bechi, Kite, and Tepi was found in ILRI14435, Gebisa, ILRI6528, respectively.

Wide genotypic range was found in CP (57 g/kg DM) and IVDMD (56 g/kg DM) (Table 3).

### 3.2 Correlation between lablab morphology and forage yield and nutritive value

The correlation between food and forage traits is presented in Table 5. The correlation between plant morphology and forage yield and nutritive value was weak (r ≤ 0.41) irrespective of the location.

**Table 5:** Correlation between forage yield and morphological traits of lablab.

	Forage yield of DM	CP	IVDMD
*Combined data*			
Forage yield	–	0.247*	0.355*
Leaf to stem ratio	−0.249*	0.107	−0.034
Leaf area	0.123*	0.323*	0.354*
Number of pods per plant	−0.163*	−0.319*	−0.329*
Plant height	0.266*	0.268*	0.34*
Number of leaves per plant	0.32*	0.027	−0.028
Number of branches per plant	0.185*	0.-0.026	0.144*
*Bechi*			
Forage yield	–	−0.023	0.053
Leaf to stem ratio	0.058	0.151	0.029
Leaf area	−0.031	0.238	0.038
Number of pods per plant	0.123	0.284	0.351*
Plant height	−0.034	0.143	0.024
Number of leaves per plant	−0.011	0.059	0.083
Number of branches per plant	0.103	−0.013	0.121
*Kite*			
Forage yield	–	−0.309*	−0.388*
Leaf to stem ratio	−0.084	0.008	0.117
Leaf area	0.3	−0.249	−0.027
Number of pods per plant	0.452	−0.381*	−0.202
Plant height	0.398*	−0.195	−0.106
Number of leaves per plant	0.692*	−0.181	−0.275
Number of branches per plant	−0.311	0.24	0.218
*Tepi*			
Forage yield	–	0.103	−0.243
Leaf to stem ratio	−0.381*	−0.16	0.089
Leaf area	0.143	−0.13	0.098
Number of pods per plant	0.182	0.073	−0.164
Plant height	−0.123	−0.01	−0.086
Number of leaves per plant	0.042	−0.058	−0.128
Number of branches per plant	0.301	−0.408*	−0.307

DM = dry matter; CP = crude protein; IVDMD = *in vitro* dry matter digestibility; * = P ≤ 0.05.

## 4 Discussion

### 4.1 Variability in forage yield and nutritive value

One goal of the goal of the current study is to assess the genetic and environmental variation in forage yield and nutritive value of lablab.

The genotypic variation in forage yield and nutritive value of lablab within a given location was reported in the current study. In general, our results showed that Kite had the lowest forage yield of DM compared to the other locations. This maybe because its soil had the lowest pH, P, K, Ca and Mg.

A total of 79 g of dietary crude protein is required by a cow to produce one kg 4% fat milk [26]. Accordingly, ignoring the genotypic variability when recommending lablab genotype might result in huge loss in biomass and nutrients (could be translated into 32.5 t milk/ha, 7.75 t milk/ha and 34 t milk/h in Bechi, Kite, and Tepi).

Additionally, our study showed that changing the planting location of ILRI11613 from Tepi to Bechi caused a huge reduction in forage yield of dry matter by 11.1 t DM/ha. Changing the location of planting ILRI11613 genotype from Tepi to Kite would result in huge change of CP yield (3.35 t CP/ha/year) which would be converted into 34.6 t milk/year. In other words, recommendation of lablab improved genotypes without considering the geographical location (where the genotype perform the best) would limit the farming plot from getting its maximum potential of lablab forage production. The wide genotypic variation in forage yield and nutritive value would help extension workers jointly with farmers to select lablabe genotypes which maximise farming unit outcome of forage in mixed farming systems of Ethiopia.

The wide genetic variation in grain yield, forage yield and forage nutritive value in lablab agrees with the results reported in cereal and legume crops like millet [27,28], maize [29] lentil [30], chickpea [31] and faba bean [32].

The significant effect of genotype-location interaction on forage yield and nutritive value of lablab means that the relative ranking of lablab genotypes for forage yield and nutritive value is not the same across different environments. The interaction between genotype and location in lablab forage yield and nutritive value traits is in line with [29] findings on maize [29], lentil [30] and chickpea [31].

The effect of location on the relative ranking of lablab varieties for forage yield of lablab should be included in formal agricultural extension approach in the mixed farming system. That would decrease the chance of disagreement between farmers and both national and international research centres about the relative ranking of lablab varieties and improve the uptake of improved varieties of lablab in mixed farming systems.

### 4.2 Correlation between lablab morphology and forage yield and nutritive value

Identifying forage yield and nutritive value of lablab is extremely important to have better understanding of feed inventory and make more informed decision on livestock feeding in the farm. in addition to that, forage yield and nutritive value are primer criteria in all lablab improvement programs.

The current study showed a weak correlation between morphology and yield nutritive value (CP and IVDMD) of lablab forage in all locations. That means morphology cannot explain the variability in yield and nutritive value of lablab forage. Thus, yield and nutritive value of lablab forage should be measured directly using conventional methods. Our results agree with [33] where morphological traits were unable alone to predict nutritive value of pearl millet straw. Although, strong association between morphology and forage yield and nutritive value was reported in rice [34,35] and faba bean [32], the conclusions of these studies are not robust since they were based on results of single environment trials.

Farmers in mixed farming systems use morphology to rank crop varieties for straw yield and nutritive value (Mulugeta et al., unpublished data). Majority of farmers associate straw yield with plant height and nutritive value with ratio of leaf to the overall straw biomass (Keno et al., -unpublished data). They might be driven by their traditional knowledge or by extension workers who seem to extrapolate the results where morphology correlated strongly with forage yield and nutritive value (i.e. in rice [34,35] and faba bean [32]) to lablab (Wamatu et al., 2023- personal communication).

The current study showed that ranking lablab varieties for forage yield by farmers dose not match that of lablab breeders. Accordingly, breeders and farmers most likely to disagree over yield and nutritive value of lablab genotypes for different locations. Consequently, there is a high chance that farmers in mixed farming systems would reject new lablab genotypes recommended by breeders.

Introducing high yielding forage crops (like lablab) to the mixed farming systems was suggested as a strategy to promote sustainable agricultural production by improving nutrients supply to livestock and an alternative to crop residue which could be used for soil mulching [36,37]. However, the adoption rate of lablab production in mixed farming systems of Ethiopia is still low due to many socioeconomic factors ([12,13], and [38]). Thus, the independent relation between morphology and forage yield and nutritive value of lablab proved by the current study should be shared widely with farmers in mixed farming systems via formal and informal channels to avoid such wrong extrapolation and therefore to enhance uptake of improved lablab varieties.

Enhancing the adoption rate of improved lablab varieties in mixed farming systems would increase nutrients supply to livestock for more meat and milk production. It would also minimise the use of crop residues for livestock feeding leading to more crop residue biomass left in the cropping plots for mulching, which would decrease soil deterioration in productivity in the mixed farming system [39]. However, animal numbers, intensity of land use, sale value, and other factors influence what crop residue is used for.

## 5 Conclusion

Farmers should consider both the genotype and location when they make decision about selecting lablab genotype. Morphological traits of lablab cannot be used to determine forage yield and nutritive value. The results of the current study should be shared with farmers through formal and informal extension to improve the adoption of improved lablab genotypes in mixed farming systems.

## Funding statement

The research is financially supported by the 10.13039/501100011687Ethiopian agricultural research institute.

## Author contribution statement

Melekam Aleme; Metekia Tamiru: Conceived and designed the experiments; Performed the experiments; Analyzed and interpreted the data; Contributed reagents, materials, analysis tools or data; Wrote the paper.

Ashraf Alkhtib; Getnet Assefa; Aemiro Kehaliew; Gezahagn Mengistu: Conceived and designed the experiments; Analyzed and interpreted the data; Wrote the paper.

Taye Tolemariam: Conceived and designed the experiments; Analyzed and interpreted the data; Contributed reagents, materials, analysis tools or data; Wrote the paper.

Emily Burton; Geert Janssens: Analyzed and interpreted the data.

## Data availability statement

Data will be made available on request.

## Additional information

No additional information is available for this paper.

## Declaration of competing interest

The authors declare that they have no known competing financial interests or personal relationships that could have appeared to influence the work reported in this paper.

## References

[1] Ryschawy J., Choisis N., Choisis J., Joannon A., Gibon A. (2012). Mixed crop-livestock systems: an economic and environmental-friendly way of farming?. Animal.

[2] Smith J., Naazie A., Larbi A., Agyemang K., Tarawali S. (1997). Integrated crop-livestock systems in sub-Saharan Africa: an option or an imperative?. Outlook Agric..

[3] Pretty J., Toulmin C., Williams S. (2011). Sustainable intensification in African agriculture. Int. J. Agric. Sustain..

[4] Ewansiha S., Chiezey U., Tarawali S., Iwuafor E. (2007). Potential of lablab purpureus accessions for crop-livestock production in the West African savanna. J. Agric. Sci..

[5] Heuzé V., Tran G., Sauvant D., Renaudeau D., Bastianelli D., Lebas F. (2016). Lablab (lablab purpureus), feedipedia, a programme by INRA. https://www.feedipedia.org/node/297.

[6] Murphy A., Colucci P. (1999).

[7] FAO (2014).

[8] Abule E., Umunna N., Nsahlai I., Osuji P., Alemu Y. (1995). The effect of supplementing teff (Eragrostis tef) straw with graded levels of cowpea (Vigna unguiculata) and lablab (Lablab purpureus) hays on degradation, rumen particulate passage and intake by crossbred (Friesian × Boran (zebu)) calves. Livest. Prod. Sci..

[9] Nyambati E., Sollenberger L., Eilitta M., Mureithi J. (2009). Residual effects of relay-cropped mucuna and lablab on maize and bean yields in northwest Kenya. Afr. J. Agric. Res..

[10] Tibayungwa F., Mugisha J., Nabasirye M. (2011). Modelling the effect of supplementing elephant grass with lablab and desmodium on weight gain of dairy heifers under stall-feeding system. Afr. J. Agric. Res..

[11] Ajayi F., Babayemi O., Taiwo A. (2008). Effects of supplementation of Panicum maximum with four herbaceous forage legumes on performance, nutrient digestibility and nitrogen balance in West African dwarf goats. Anim. Sci. J..

[12] Minde J.J., Venkataramana P.B., Matemu A.O. (2020).

[13] Letting F.K., Venkataramana P.B., Ndakidemi P.A. (2021). Breeding potential of lablab [Lablab purpureus (L.) Sweet]: a review on characterization and bruchid studies towards improved production and utilization in Africa. Genet. Resour. Crop Evol..

[14] Muktar M., Sartie A., Negawo A., Habte E., Jones C. (2021). The Joint XXIV International Grassland and XI Rangeland 2021 Congress.

[15] Ewansiha S.U., Chiezey U.F., Tarawali S.A., Iwuafor E.N.O. (2007). Morpho-phenological variation in Lablab purpureus. Trop. Grassl..

[16] Kahsay R., Mekuriaw Y., Asmare B. (2021).

[17] Hassan M.R., Amodu J.T., Muhammad I.R., Jokthan G.E., Abdu S.B., Abdullahi B., Adamu H.Y., Musa A., Sani I., Akpensuen T.T. (2014). Forage yield and quality of lablab (lablab purpureus L. Sweet) intercropped with maize (Zea mays L.) with flooded irrigation system in the semi-arid zone of Nigeria. J. Agric. Sci..

[18] Adem A., Mekuriaw Y., Asmare B. (2021). Effects of accessions and fertilizer levels on agronomic characteristics, forage biomass yield and nutritive value of lablab (lablab purpureus L) under irrigation in dry lands of Ethiopia. Cogent Food Agric..

[19] Aleme M. (2022). Performance evaluation of lablab genotypes across various locations of Ethiopia. Advances in Agriculture.

[20] Alkhtib A., Wamatu J., Wegi T., Rischkowsky B. (2016). International Conference on Pulses.

[21] Kafilzadeh F., Maleki E. (2012). Chemical composition, in vitro digestibility and gas production of straws from different varieties and accessions of chickpea. J. Anim. Physiol. Anim. Nutr..

[22] Aoac (2003).

[23] Van Soest P., Robertson J., Lewis B. (1991). Methods for dietary fiber, neutral detergent fiber, and nonstarch polysaccharides in relation to animal nutrition. Journal of Dairy Sceince.

[24] Tilley J., Terry R. (1963). A two-stage technique for the in vitro digestion of forage crops. Grass Forage Sci..

[25] R core Team R. (2017).

[26] Kearl L.C. (1982).

[27] Bidinger F.R., Blümmel M., Hash C.T., Choudhary S. (2010). Genetic enhancement for superior food-feed traits in a pearl millet (Pennisetum glaucum (L.) R. Br.) variety by recurrent selection. Anim. Nutr. Feed Technol..

[28] Blümmel M., Bidinger F., Hash T. (2007). Management and cultivar effects on ruminant nutritional quality of pearl millet (Pennisetum glaucum (L.) R. Br.) stover: II. Effects of cultivar choice on stover quality and productivity. Field Crops Res..

[29] Ertiro B.T., Twumasi-Afriyie S., Blümmel M., Friesen D., Negera D., Worku M., Abakemal D., Kitenge K. (2013). Genetic variability of maize stover quality and the potential for genetic improvement of fodder value. Field Crops Res..

[30] Alkhtib A., Wamatu J., Ejeta T., Rischkowsky B. (2017). Integrating straw yield and quality into multi-dimensional improvement of lentil (Lens culinaris). J. Sci. Food Agric..

[31] Wamatu J., Alemu T., Tolera A., Beyan M., Alkhtib A., Eshete M., Ahmed S., Rischkowsky B. (2017). Selecting for food-feed traits in desi and kabuli genotypes of chickpea(Cicer arietinum). J Exp Bio AgriSci.

[32] Alkhtib A., Wamatu J., Wegi T., Rischkowsky B. (2016). Variation in the straw traits of morphological fractions of faba bean (Vicia faba L.) and implications for selecting for food-feed varieties. Anim. Feed Sci. Technol..

[33] Ravi D., Anandan S., Khan A., Bidinger F., Nepolean T., Hash C., Blümmel M. (2010). Morphological, chemical and in vitro traits for prediction of stover quality in pearl millet for use in multidimensional crop improvement. Anim. Nutr. Feed Technol..

[34] Vadiveloo J. (1995). Factors contributing to varietal differences in the nutritive value of rice straw. Anim. Feed Sci. Technol..

[35] Grev A.M., Wells M.S., Catalano D.N., Martinson K.L., Jungers J.M., Sheaffer C.C. (2020). Stem and leaf forage nutritive value and morphology of reduced lignin alfalfa. Agron. J..

[36] Jaleta M., Kassie M., Shiferaw B. (2013). Tradeoffs in crop residue utilization in mixed crop-livestock systems and implications for conservation agriculture. Agric. Syst..

[37] Alkhtib A., Wamatu J., Kassie G., Rischkowsky B. (2017).

[38] Mekoya A., Oosting S., Fernadez-Rivera S. (2006). Book of Abstracts of the 57th Annual Meeting of the European Association for Animal Production.

[39] Keno M.T., Wamatu J., Alkhtib A., Tolemariam T., Demeke S., Janssens G.P.J. (2021). Barley straw use for animal feed and soil mulch in Ethiopian highlands mixed crop-livestock systems. Sustainability.

